# Incidence of Infective Endocarditis in Dogs After Balloon Dilatation of Congenital Pulmonic Stenosis Without Peri- and Postoperative Antimicrobial Prophylaxis

**DOI:** 10.3390/ani16162629

**Published:** 2026-08-21

**Authors:** Daphne I. Zeedijk, Viktor Szatmári

**Affiliations:** Department of Clinical Sciences, Faculty of Veterinary Medicine, Utrecht University, Yalelaan 108, 3584 CM Utrecht, The Netherlands

**Keywords:** antibiotic, antimicrobial stewardship, bacterial endocarditis, congenital heart disease, catheter intervention, pulmonary valve, sepsis, surgical site infection

## Abstract

Balloon valvuloplasty is a commonly performed catheter-based procedure in dogs with congenital pulmonic stenosis. Worldwide, veterinary cardiologists often administer peri- and postoperative prophylactic antimicrobials to prevent infectious complications, like bacterial endocarditis. Infective endocarditis of the pulmonary valve is, however, extremely rare in dogs, and it has never been reported after balloon valvuloplasty. The objective of this retrospective case series was to investigate the incidence of infective endocarditis, if balloon dilatation of pulmonic stenosis was performed without antimicrobial prophylaxis. Medical records of dogs operated at a university teaching hospital in a 20-year period from 2006 to 2025 were reviewed for clinical signs and echocardiographic changes that could be caused by infective endocarditis. Of the 83 dogs that were operated without peri- or postoperative antimicrobial prophylaxis, survived the surgery, and had at least three weeks of follow-up, none developed infective endocarditis. Follow-up in 68 dogs was based on medical history, physical examination and echocardiography during rechecks at the authors’ institution. Follow-up information from the remaining 15 dogs was gained from the owners and/or the referring veterinarians. Our study demonstrated zero event rate in dogs that underwent balloon dilatation of congenital pulmonic stenosis without antimicrobial prophylaxis.

## Simple Summary

One of the most commonly performed minimally invasive catheter-based cardiac surgeries in dogs is balloon dilatation of the congenitally fused valves of the pulmonary artery. Though this is a clean surgery, many cardiologists tend to administer prophylactic antimicrobial drugs during and after surgery in order to prevent infectious complications. In the fight against antimicrobial resistance, reduction of antibiotic use is an important measure. In our study we retrospectively looked for possible infections in dogs that underwent this procedure in a 20-year period. None of the 83 dogs that received no peri- or postoperative prophylactic antimicrobial drugs developed clinical signs or echocardiographic changes that could be compatible with bacterial infection of the heart valves. We conclude that routine use of perioperative prophylactic antimicrobials is not justified for this minimally invasive catheter-based heart surgery.

## 1. Introduction

Pulmonary valve stenosis is one of the three most commonly encountered congenital heart diseases in dogs [1,2,3,4,5,6,7]. Increased cardiac morbidity and mortality have been described in dogs with severe stenosis, i.e., a Doppler-derived transpulmonary pressure gradient exceeding 80 mmHg [4,5,6,7]. Balloon valvuloplasty is currently the first-choice treatment for most dogs with severe pulmonary valve stenosis [4,5,6,7]. This minimally invasive catheter-based interventional procedure, performed via the right femoral or the right jugular vein, increases the pulmonary valvular orifice by tearing the congenitally malformed, fused valves [5,6,7]. Although balloon valvuloplasty is a relatively safe procedure, and it is performed routinely by veterinary cardiologists all over the world, the procedure is not without risks [5,6,7,8,9,10]. While transient systemic arterial hypotension and supraventricular or ventricular arrhythmias are very common complications, cardiac perforation, pulmonary artery dissection, “suicide” right ventricle, cardiac arrest, and coronary artery damage are rare [5,6,7,8,9,10]. While these complications can in some dogs be well tolerated, in others they could be fatal [5,6,7,8,9,10]. A theoretical complication of balloon valvuloplasty is infective endocarditis of the mechanically damaged valve, since both prerequisites for bacterial colonization, i.e., endothelial disruption and bacteriemia, might be simultaneously present [11]. Though endocardial damage is a known risk factor for the development of infective endocarditis [11], bacterial endocarditis of the pulmonary valve is exceedingly rare with only two confirmed and two suspected cases reported in the veterinary literature [12,13,14]. However, a recently published survey among veterinary cardiologists showed that 105 of the 139 (76%) respondents routinely use perioperative (i.e., intravenous) and 25 (18%) also postoperative (i.e., oral) antimicrobial prophylaxis for a median duration of 7 days with a range of 2–10 days [15]. This frequent and prolonged use of antimicrobial prophylaxis in dogs is surprising, given that international human medical guidelines do not recommend the use of prophylactic antimicrobials for catheterization procedures without implants [16,17,18]. With antimicrobial resistance becoming an increasing worldwide concern, it is essential that veterinarians reflect on the broader impact of their daily clinical choices and work towards optimizing the balance between patient safety and responsible antibiotic stewardship [18,19,20]. Though species differences between dogs and humans might limit the applicability of human antimicrobial prophylaxis guidelines for cardiac interventions, current veterinary guidelines strongly recommend against both peri- and postoperative antimicrobial prophylaxis in clean and also clean-contaminated soft tissue surgical procedures [21,22,23].

This retrospective single-center cohort study aimed to investigate the incidence of clinically apparent infective endocarditis of the pulmonary valve in dogs that underwent balloon valvuloplasty of a congenital pulmonary valve stenosis or double-chambered right ventricle without peri- or postoperative antimicrobial prophylaxis in the authors’ institution.

## 2. Materials and Methods

### 2.1. Animals

Case records of client-owned dogs that underwent balloon valvuloplasty of pulmonic stenosis or balloon dilatation of a double-chambered right ventricle at the Cardiology Service of the Companion Animal Clinic of Utrecht University between August 2006 and December 2025 were searched in the electronic database and physical archive kept at the facility. Dogs with an incomplete or missing anesthesia or surgery report, or dogs with insufficient follow-up information (<3 weeks postoperative) were not included. Dogs that underwent a cardiac catheterization procedure of the right heart without introduction or inflation of a balloon catheter were not included. Dogs that received peri- or postoperative antimicrobial prophylaxis were not included in the analysis, but their results are presented separately.

### 2.2. Surgical Procedure

Diagnosis of severe pulmonary valve stenosis was made by the presence of a loud systolic murmur with the point of maximal intensity in the left cardiac base on auscultation, followed by a comprehensive echocardiogram using 2-dimensional, color, pulsed- and continuous-wave Doppler modes [2,4,5,6,7,24].

On the day of the procedure, the dogs underwent a pre-anesthetic physical examination including a short history taking from the owner.

All procedures took place in the fluoroscopy unit, which is not a dedicated surgical theater. This means that the room did not have a positive air pressure ventilation system, i.e., higher air pressure in the room than in the adjoining corridors, and it is categorized as a “contaminated” area in surgical terms. 

The cardiac catheterization procedure was performed under general anesthesia. The anesthesia protocol was formulated by the responsible anesthesiologist and adapted to the individual needs of the patient. The protocol underwent several adjustments during the two decades. All dogs were premedicated intramuscularly, recently using medetomidine combined with either butorphanol or methadone, and the anesthesia was induced with propofol intravenously. After anesthetic induction, all patients were intubated. The anesthesia was maintained via inhalation isoflurane vaporized in oxygen. The dogs were mechanically ventilated, and analgesia included intravenous continuous-rate infusions of fentanyl, medetomidine/dexmedetomidine or ketamine. Although cardiac catheterization is a minimally invasive procedure, cut-down to the vein and cannulation of vessels themselves can still lead to a sympathetic response, which warrants a continuous-rate infusion of an analgesic drug during this procedure (e.g., ketamine). An additional benefit of such drugs is the sparring effect of isoflurane minimal alveolar concentration (MAC), to reduce the unwanted cardio-respiratory effects of high concentrations of isoflurane (e.g., vasodilation). During the procedure, the dogs were continuously monitored using an electrocardiogram, pulse-oximetry, rectal temperature, end tidal CO_2_ concentration, and arterial blood pressure via either indirect oscillometric method or directly through intra-arterial measurement via either the femoral, metatarsal, metacarpal, or median coccygeal artery.

Peri- and/or postoperative antibiotics were only administered when the anesthesiologist decided to give them, or when an additional surgical procedure was performed on the same day under the same general anesthesia session, and the responsible surgeon asked for them.

For the balloon dilatation the dogs were positioned on the fluoroscopy table under general anesthesia in left or right lateral recumbency, depending on the vein used for vascular access: left lateral recumbency for right jugular venous access and right lateral recumbency for right femoral venous access. After aseptic preparation of the skin (i.e., shaving and scrubbing), venous access was obtained either via surgical cut-down (femoral or jugular vein) or via percutaneous puncture (jugular vein) using the modified Seldinger’s technique [5,6,7]. In case of surgical cut-down the peripheral end of the vein was ligated with absorbable multifilament suture material (Polyglactin, Vicryl Plus 2-0, 3-0 or 4-0, ETHICON, Johnson & Johnson International, Diegem, Belgium). In the central end of the vein (proximal in case of femoral vein and caudal in case of the jugular vein), a short (5–13 cm depending on the access site) introducer with a hemostatic valve was placed and secured to the skin with a single stitch. After placing the introducer, an angiographic catheter—either an angled pigtail catheter, a Berman angiographic balloon catheter, a multifunctional catheter or a right coronary catheter, depending on the size of the dog and the year—was placed through the introducer in the lumen of the right ventricle to perform a ventriculogram. The right ventriculogram was performed in order to (1) localize the pulmonic valve, (2) visualize the valve morphology, (3) measure the diameter of the pulmonary valve annulus, (4) visualize the pulmonary artery and the magnitude of post-stenotic dilation, and (5) gain information on the potential presence of a pre-pulmonary coronary artery anatomy in the levophase. For the right ventriculogram, iodinated radiographic contrast agent was injected with a contrast pump (approximately 1 mL/kg with the highest pressure that the label of the angiographic catheter indicated). After removing the angiographic catheter, an end-hole catheter—either a balloon wedge catheter or a multifunctional angiographic catheter, with or without a (hydrophilic of standard exchange) guidewire—was used to cross the tricuspid and pulmonary valves. In each chamber the blood pressure was measured directly through the diagnostic catheter: the right atrium, right ventricle, and pulmonary artery.

Once the tip of the catheter was placed in a peripheral pulmonary artery branch, a stiffer non-hydrophilic guidewire was inserted through its lumen to a stable wedged position. Depending on the size of the dog, the guidewire stiffness varied; in small dogs (about <8 kg) a straight-tip standard exchange wire of 0.025- or 0.035-inch thickness was used, in medium-sized dogs (approximately 8–20 kg) either a straight or a J-tip Rosen or Amplatz super-stiff guidewire of 0.035-inch thickness was inserted, and in large dogs (about >20 kg) a straight-tip extra-stiff guidewire of 0.035-inch thickness was used (from various brands and manufacturers, such as Terumo Europe, Leuven, Belgium; Cordis Europa N.V., Amersfoort, the Netherlands; and Cook Nederland B.V. Amsterdam, the Netherlands. Subsequently, the catheter was removed over the guidewire. Balloon diameter was chosen based on measurements of the pulmonary annulus obtained during the preoperative transthoracic 2-dimensional echocardiogram and the right ventriculogram, and a 30–50% oversizing was aimed. The length of the inflated balloon varied between 3 and 8 cm depending on the size of the dog and the available anatomical length from the right ventricular apex to the pulmonary bifurcation measured on the echocardiographic and angiographic images. The balloon catheter was introduced over the guidewire to the appropriate position and was inflated with a mixture (1:3–4 dilution) of iodinated radiographic contrast material and physiologic saline solution using either a syringe (20 or 50 mL depending on the balloon size) or a dedicated inflation device with a manometer (depending on the year and the balloon size, 20–60 mL, from various manufacturers). Various types of balloon valvuloplasty catheters were used, from the more compliant small-profile balloons (such as Tyshak II, Heart Medical, Best, Netherlands, or VACS II, Osypka, Rheinfelden, Germany) to high-pressure balloon catheters (such Armada 35 balloon dilatation catheter, Abbott Cardiovascular, Heerlen, Netherlands, or Atlas Gold PTA-balloon, Bard Peripheral Vascular, Tempe, AZ, United States). The valvuloplasty balloon was inflated once or multiple times to its maximal diameter (using visual inspection and in case an inflation device was used also the manometer to reach the nominal pressure without exceeding the burst pressure), and then it was quickly emptied. Thereafter, the balloon catheter was removed over the guidewire, and the end-hole angiographic catheter was inserted once again after removal of the guidewire, to measure the pressures in the pulmonary artery, right ventricle, and right atrium, while pulling the catheter back. Finally, the angiographic catheter and the introducer were removed from the access vein. Both ends of the vein were ligated in case of surgical cut-down with absorbable multifilament suture material (Vicryl). In case of percutaneous puncture, a purse string suture was placed around the skin wound secured with a 3-way stopcock, which was removed by the owner the following day. For surgical cut-down, the subcutaneous tissue was also closed with absorbable multifilament or monofilament suture material (Polyglactin Vicryl Plus 3-0 or 4-0, or Poliglecaprone, Monocryl Plus, 2-0, 3-0 or 4-0, ETHICON, Johnson & Johnson International, European Logistics Centre, Diegem, Belgium) in two layers, and the skin was closed with either absorbable or nonabsorbable monofilament suture material (Poliglecaprone, Monocryl Plus or poliamid, Ethilon 3-0 or 4-0 ETHICON, Johnson & Johnson International, European Logistics Centre, Diegem, Belgium or Ethilon). No covering bandage was applied, only wound spray was used (Opsite, Smith & Nephew, Hoofddorp, The Netherlands). The skin sutures were removed 10–14 days after surgery by the referring veterinarian.

All disposable materials such as diagnostic catheters, guidewires, balloon valvuloplasty catheters and inflation devices were in their original sterile package. No used and re-sterilized materials were used. No heparin was used during the procedures.

Dogs were discharged from the clinic the same day as the procedure, if no complications took place. Postoperative pain relief was provided with 3–4 days of oral non-steroidal anti-inflammatory drugs (meloxicam for small dogs and carprofen for larger dogs) in case of surgical cut-down. No postoperative medication was given in case of percutaneous jugular venous access.

### 2.3. Follow-Up

Recheck examination, including history taking, physical examination and echocardiography, was recommended at the authors’ clinic at least two months after the procedure.

If the owner failed to return for this recheck examination, or the examination took place less than three weeks after surgery, a follow-up telephone call took place to gain information about the postoperative period. When owners could not be reached by phone or email, the referring veterinarian was contacted to provide medical information on the postoperative period.

To determine if dogs had possibly developed infective endocarditis within three weeks after balloon valvuloplasty, records of the follow-up consultations were reviewed for the presence of clinical signs that could potentially be compatible with a bacterial endocarditis. These clinical signs and abnormal findings included lethargy, anorexia, lameness, and fever [11,12,13,14]. In cases where echocardiogram was performed at least three weeks after the procedure, the echocardiogram reports were reviewed for valvular vegetations or erosive lesions on the valves [11,12,13,14]. When owners were contacted by telephone, they were asked if their dog showed any, or specifically the above-described, clinical signs in the first three months after the procedure. The owners were asked the following questions: (1) How was the dog doing in the first three postoperative months? (2) Have you visited your veterinarian for any reason during in this period? If yes, why? (3) Have you noticed anything abnormal in the general functioning of your dog within three months after surgery, such as lethargy, decreased appetite, or changes in endurance, water consumption, urination or defecation? 

When the referring veterinarian was contacted for follow-up information, their patient files were reviewed for similar findings noted in the three-week postoperative period.

No routine blood tests, such as complete blood count, inflammatory markers (such as serum C-reactive protein level) or blood culture, were performed, if there was no clinical indication for a septic process based on the history, physical examination and echocardiographic findings.

### 2.4. Statistics

Commercially available statistical software (Excel version 365; Microsoft Corporation, Redmond, WA, USA) was used for data analysis. Median values were calculated. Results are presented as median and range.

## 3. Results

### 3.1. Dogs Without Antimicrobial Prophylaxis

**Figure 1:**
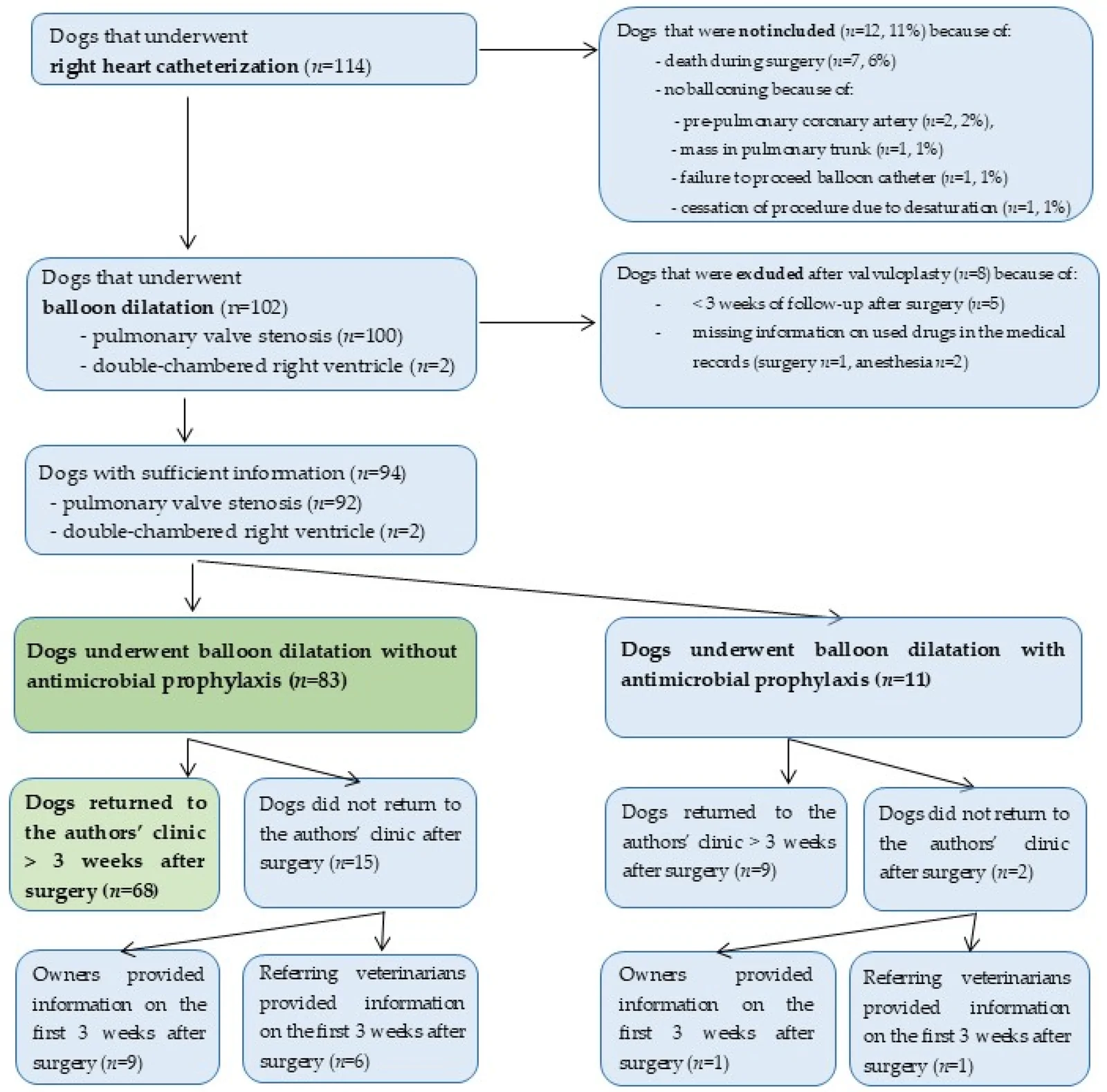
Inclusion and exclusion reasons of dogs that underwent right heart catheterization. The final study group is indicated within the box with a green background and bold letter type.

In the 20-year period, 114 dogs underwent a right heart catheterization procedure to attempt balloon dilatation of a pulmonic stenosis or double-chambered right ventricle. Of these 114 dogs, 102 dogs actually underwent the balloon dilatation procedure and survived the surgery, 100 dogs for pulmonary valve stenosis and two dogs for double-chambered right ventricle. The reasons why twelve dogs were not included were death during the procedure (*n* = 7), and not performing a balloon dilatation for one of the following reasons: inability to cross the stenosis (*n* = 2), pre-pulmonary coronary artery (*n* = 2), or acquired pulmonic stenosis (*n* = 1) (Figure 1).

The median age of the dogs on the day of surgery was 213 days (7 months, range: 82 days–2460 days [6.7 years]), and the median body weight was 16.2 kg (range: 1.5–56 kg). Of the 39 represented breeds, French bulldog was the most common (*n* = 17, 18%). Most dogs (*n* = 90, 96%) were thought to have a valvular stenosis. The preoperative Doppler-derived pressure gradient in awake, non-sedated dogs was above 80 mmHg in 90% (*n* = 85) of the dogs, with a median of 114 mmHg (range 61–230 mmHg). Median surgery time was 114 min (range: 55–323 min). The median reduction in pressure gradient after balloon valvuloplasty, measured invasively intraoperatively, was 69% (range: 3–100%).

All dogs were alert and normothermic during the preanesthetic examination on the day of surgery.

Of the 102 dogs, eight dogs were excluded because medical information on the surgery in the surgery report (*n* = 1) or drugs used during the surgery in the anesthesia report (*n* = 2) was missing, or insufficient follow-up information (i.e., less than three weeks) was available (*n* = 5). Of the remaining 94 dogs, 83 dogs received no peri- and/or postoperative antimicrobials; these dogs made up the final study group (Figure 1).

Of these 83 dogs, none were suspected to have or were diagnosed with infective endocarditis. The event rate was 0% (95% confidence interval 0.0–3.5%). Of the 83 dogs, 68 were evaluated during a recheck examination, including an echocardiogram at the authors’ institution at least three weeks after the procedure (median 88 days, range 22–2198 days [6 years]). The median Doppler-derived pressure gradient was 55 mmHg. For the remaining 15 dogs, the medical information on the three-week follow-up period was missing because they did not return to the authors’ institution. Follow-up information was provided by the owners for nine dogs and by the referring veterinarian for six dogs.

### 3.2. Dogs with Antimicrobial Prophylaxis

None of the eleven dogs that received prophylactic antimicrobial drugs on the day of surgery and/or thereafter developed signs of a possible endocarditis (Figure 1). Of these eleven dogs, nine returned for a recheck and were examined at the authors’ institution. Follow-up information was provided by the owners for one dog and by the referring veterinarian for the other dogs. Of the eleven dogs that were treated with antimicrobial drugs, six dogs received perioperative antimicrobial prophylaxis from the attending anesthesiologist without a request by the attending cardiologist (a single dose of 20 mg/kg intravenous cefazolin in three dogs and a single dose of 20 mg/kg intravenous amoxicillin with clavulanic acid was given to the other three dogs, of which one dog received oral amoxicillin with clavulanic acid for five days); two dogs because of hemorrhage from the femoral venous access site (one a single intravenously administered dose of cefazolin, and the other amoxicillin with clavulanic acid); one dog only received postoperative oral amoxicillin with clavulanic acid because of post-surgical aspiration pneumonia for 14 days; and two dogs because of additional elective surgeries on the same day, of which one underwent staphylectomy and alar fold correction (a single intravenous dose of 20 mg/kg cefazolin), and the other dog had castration and removal of the first digit from the hindlegs (a single dose of intravenous 20 mg/kg cefazolin followed by 10 days of oral amoxicillin and clavulanic acid). One dog developed fever a few days after the procedure, which was treated with oral antibiotics by the referring veterinarian. The fever resolved within a couple of days, and recheck echocardiography at the authors’ institution three months postoperatively revealed no abnormalities of the heart valves that could be compatible with infective endocarditis.

## 4. Discussion

In this retrospective case series, we have shown that none of the 83 dogs that underwent a transcatheter balloon dilatation of a congenital pulmonary valve stenosis or a double-chambered right ventricle without peri- and postoperative prophylactic antimicrobial administration at our academic teaching hospital developed infective endocarditis within three weeks after the procedure.

Infective endocarditis can occur when endocardial damage and bacteremia are simultaneously present [11,14,17,18,25]. Endocardial damage can be a result of mechanical damage, like is the case during cardiac catheterization procedures, when guidewires and catheters inadvertently, or the valvuloplasty balloon catheter purposefully, damage the valve [5,6,7]. When endothelial disruption occurs, extracellular matrix proteins and thromboplastin initiate the coagulation cascade, resulting in the formation of thrombus on the damaged endothelium [11,25]. This thrombus readily binds bacteria if bacteremia is present [11]. Evidence from human studies suggests a direct relationship between the normal resting pressure on closed heart valves and the incidence of valvular endocarditis: valves that experience high resting pressures may be more susceptible to scarring and bacteria may be more easily forced into the endothelium of these valves [26]. This translates to the fact that aortic and mitral valves are the most susceptible, whereas the pulmonary valve is the least susceptible to developing an infective endocarditis [11,14,25,26]. In clinical cases of canine infective endocarditis, conditions such as prostatitis, pyelonephritis or dental disease may act as potential sources of bacteremia; however, in many cases, the primary site of bacterial entry into the bloodstream remains unknown [11,14,25]. In dogs that undergo surgery, the surgical wound may be the porte d’entrée for the bacteria.

There are no clear cut-off values known regarding the duration of incubation period for the development of septic complication, such as infective endocarditis, after a clean non-contaminated elective soft tissue surgery that does not leave any foreign material behind, such as balloon dilatation. A study investigating the incubation period of infective endocarditis in humans found that 78% of cases developed within 7 days of the exposure to bacteria and 85% within 14 days [27]. Median duration of illness was 10 days (range 0–390 days) in a study on 68 dogs with naturally occurring endocarditis [14]. An additional reason for choosing three weeks of follow up period in our study was that this was the longest duration of postoperative antimicrobial prophylaxis that veterinary cardiologists reported to administer after cardiac interventions in our recent survey [15]. Therefore, we decided arbitrarily a minimum of three weeks of postoperative follow-up period.

Though dogs that underwent the balloon dilatation procedure might be theoretically at a higher risk for developing infective endocarditis, no studies to date have shown that cardiac catheterization procedures are associated with the development of endocarditis, nor in dogs, or in children or in adult humans. Spontaneous occurrence of infective endocarditis of the pulmonic valve in dogs has been reported in two dogs only, and it was suspected in two other dogs [12,13,14], but never following a balloon valvuloplasty procedure. This suggests that post-valvuloplasty endocarditis of the pulmonary valve must be extremely rare in dogs, if it exists at all. Infective endocarditis following balloon valvuloplasty of the pulmonary valve is also extremely rare in humans, where a study described a single case out of 73 performed surgeries [28]. Human medical guidelines recommend no antimicrobial prophylaxis for balloon valvuloplasty [16,17].

Definitive diagnosis of infective endocarditis can only be made with histopathological examination of the heart [11,12,13,14,25,29,30]. Clinical signs of infective endocarditis in dogs are non-specific, and most commonly consist of lethargy, inappetence, shifting lameness, and reluctance to move [14,25]. On physical examination most dogs with endocarditis have (recurrent) fever, a new or changing heart murmur, various arrhythmias and consequences of a thromboembolic event [11,12,13,14,25,29,30]. A diastolic murmur is particularly suggestive of infective endocarditis, as this is often the result of an aortic valve regurgitation [11,12,13,14,25,29,30]. Large-breed dogs older than 5 years and those with a subaortic stenosis are predisposed [11,12,13,14,25,29,30]. Though diagnosing infective endocarditis both in living humans and in dogs can be challenging, it is a potentially life-threatening condition with high morbidity and mortality that causes obvious, and in most cases severe, clinical signs with fast deterioration [11,12,13,14,25,29,30]. That is probably the reason why many veterinary cardiologists are reluctant to perform cardiac catheterization procedures without antimicrobial prophylaxis, because they are worried of this devastating and often fatal complication [15]. Among the diagnostic criteria of infective endocarditis, the echocardiographic findings of vegetative valvular lesions are the most specific both in dogs and humans [11,12,13,14,29,30]. Specifically for this reason we chose echocardiography together with history taking to identify cases with potential postoperative infective endocarditis. In addition, this test is practical and had to be performed anyway to assess the long-term result of the ballon dilatation procedure.

In contrast to the recommendations of human medical guidelines, a recent survey showed that 105 of the 139 (76%) responding veterinary cardiologists routinely use prophylactic antimicrobials when performing balloon valvuloplasty in dogs with pulmonary valve stenosis [15]. Out of these 105 veterinarians, 58% used perioperative intravenous prophylaxis only on the day of the procedure; however, the remaining 42% used also postoperative oral antimicrobials with a median duration of 7 days (range: 2–10 days) [15]. Given the extremely low incidence of infective endocarditis in humans and dogs both spontaneously and following balloon valvuloplasty, routine antimicrobial prophylaxis does not seem to be justified in dogs, as the risk of selecting resistant bacterial strains does not outweigh the expected theoretical benefit. Appropriate surgical technique and the use of sterile disposable materials from the original package seem to be sufficient to prevent this theoretical complication, even if the procedure is performed in a non-dedicated operating theater, as we demonstrated in our study.

Though a prospective, placebo-controlled, randomized, double-blinded, multicenter study could give more reliable results to support this recommendation, given the very low incidence, and therefore low event rate of infective endocarditis after balloon valvuloplasty of congenital pulmonic stenosis, even with a very large number of enrolled patients insufficient power could be reached. Finding a sufficient number of participating centers with large caseloads would also be challenging because the recent survey among veterinary cardiologists suggests that many colleagues would not agree with such a study protocol [15]. Presumably because of the low incidence of infectious complications in people after balloon valvuloplasty of the pulmonary valve, no large-scale, double-blinded, placebo-controlled, multicenter randomized study has ever been performed to answer this question in humans either.

Surgical site infection is another possible complication of cardiac catheterization procedures. This aspect has not been investigated in the present study. However, because balloon valvuloplasty is performed using either a percutaneous puncture or a small skin incision of a couple of centimeters long that does not reach deeper than the subcutis, surgical site infection does not seem to be clinically relevant for this procedure. Though no studies have investigated the occurrence of surgical site infection in dogs after cardiac catheterization procedures, a number of recent studies on various clean soft tissue surgeries in dogs failed to show a benefit of antimicrobial prophylaxis [31,32,33,34,35,36]. In addition to the recently published guidelines for antimicrobial prophylaxis for surgical procedures in dogs [21,22,23], there is currently an ongoing project to develop guidelines for surgical site infection for veterinary medicine [37] similarly to human recommendations [38].

A major limitation of our study is its retrospective design and the absence of a placebo arm. Another limitation is that follow-up information for some dogs was obtained through telephone interview with the owners or referring veterinarians, without having performed a recheck echocardiography. The reliability of such data is lower, as mild clinical signs may have remained unnoticed. However, given that infective endocarditis is typically a life-threating systemic septic condition, it is unlikely that the presence of this disease was overlooked or went unnoticed by either the owners or the referring veterinarians [11,14,22,29,30]. The low number of animals (i.e., low power) and the low (i.e., zero) event rate of the target disease, infective endocarditis, in our study limit the robustness of the conclusions. On the other hand, it is hard to justify prophylactic antimicrobial use in hundreds of dogs to possibly prevent infective endocarditis in one case. The follow-up period of three weeks after the balloon dilatation might be too short to detect late-onset endocarditis. However, most Gram-positive bacteria (such as Staphylococci and Streptococci), which are expected to cause postoperative septic complications, have a short incubation period [25,26,27,28,29,30,31,32,33,34,35,36,37,38]. Another limitation of our study is that all cases were managed at a single institution, which may limit the generalizability of the findings. Other institutions might reuse disposable materials after cleaning and sterilization, which might not eliminate bacteria completely.

## 5. Conclusions

To conclude, in this case series not a single case of infective endocarditis following balloon valvuloplasty of the pulmonary valve in 83 dogs that received no peri- or postoperative antimicrobial prophylaxis was found. Peri- or postoperative antimicrobial prophylaxis did not seem to be necessary in dogs with pulmonic stenosis that underwent balloon valvuloplasty at our institution to prevent infective endocarditis or other major infectious complications. A large, prospective, placebo-controlled, randomized, double-blinded, international multicenter trial with standardized follow-up and diagnostic criteria could provide more solid data.

## Data Availability

Data are available from the first author upon reasonable request.
